# Identifying Molecular Biomarkers in Attention-Deficit/Hyperactivity Disorder (ADHD) – a Systematic Review of Literature and Appraisal of Evidence

**DOI:** 10.1192/bjo.2024.274

**Published:** 2024-08-01

**Authors:** Ayobami Yusuff

**Affiliations:** University of South Wales, Cardiff, United Kingdom

## Abstract

**Aims:**

At the core of medical diagnosis lies specific blood tests, urine analysis, microscopic and histologic examination of tissues, and as well radiological investigations that are usually confirmatory of the presence of a disease. However, the diagnosis of ADHD currently relies on reports of clinical symptoms which is usually subjective, with variable interpretations by different professionals, thus posing issues of misdiagnosis and reliability. This study set out to explore, appraise and summarize molecular biomarkers in literature over the past 30 years, which can be applied for the diagnosis of ADHD.

Attention-deficit/hyperactivity disorder (ADHD) is a common neuropsychiatric and neurobehavioral disorder that affects children and adolescents, and more recently, gaining recognition in adults. It is characterized by a pervasive pattern of inattention, hyperactivity, and impulsivity or a mixture of the three, that cuts across the individual's multiple domains of life.

**Methods:**

One-thousand articles collated across multiple sources and databases were systematically reviewed and analysed for this project. The keywords for the search criteria in the Boolean operators are “biomarkers and ADHD”, “molecular biomarkers and ADHD” and “biomarkers and ADHD and Diagnosis”.

**Results:**

5.6% of the articles from several types of studies were included in the final analysis after the inclusion and exclusion criteria were applied. The results revealed various heterogeneity across age, gender, ethnicity, medication status, comorbidities, and study type, in applying biomarkers to assist in the diagnosis of ADHD. Genetics and epigenetics studies were the most common type of molecular biomarkers studied and identified, accounting for 25% of the results. 80% of the studies analysed blood samples with a few others focusing on saliva, urine, cerebrospinal fluid, hair, and stool samples. All the studies identified focused on diagnostic biomarkers with 25% of them combining either prognostic or response-monitoring subtypes of biomarkers.

**Conclusion:**

This study identified several potential molecular biomarkers in ADHD. However, most of the results showed only associations between the findings and the diagnosis/occurrence of ADHD. It remains a scientific goal to identify a specific and reliable biomarker for ADHD to assist psychiatrists in making accurate diagnosis. Lastly, it would be pragmatic to explore other types of biomarkers such as radiologic and electro-neurologic markers; given that diagnosis is a constellation of signs and symptoms together with appropriate tests. Combining them logically would increase the specificity of diagnosis of ADHD. This study was completed in partial fulfilment of Master of Science (MSc) in Clinical Psychiatry with the University of South Wales.

